# Editorial Comment to Testicular seminoma arising from infertile testes 6 years after microdissection testicular sperm extraction

**DOI:** 10.1002/iju5.12408

**Published:** 2021-12-19

**Authors:** Yohei Sekino, Nobuyuki Hinata

**Affiliations:** ^1^ Department of Urology Graduate School of Biomedical and Health Sciences Hiroshima University Hiroshima Japan

A risk of testicular cancer is increased in men with male factor infertility compared with the general population in western countries.^1^ However, data on this risk in Asian countries are lacking. In this case report, Shimizu et al. reported a case report of testicular seminoma arising from right infertile testes 6 years after microdissection testicular sperm extraction (TESE).^2^

In the general population, the rate of testicular cancer is 1.30–4.30 per 100 000 males.^1^ In this report, the author showed that 4 of 1398 patients who presented to the infertility outpatients department developed testicular cancer. This finding indicates that the risk of testicular cancer in men with male factor infertility may be increased in Japan. Although further real‐world data in Japan are needed, clinicians should pay attention to the risk of testicular cancer when seeing patients with male infertility.

At the author’s institute, testicular samples are routinely extracted from patients to evaluate infertility grade when performing micro‐TESE. In this case report, immunohistochemistry showed that c‐kit and placetal alkaline phosphatase (PLAP) positive cells were found in right testicular samples from micro‐TESE. Contrary, such cells were not observed in the left testicular samples. These findings suggest the presence of germ cell neoplasia *in situ* (GCNIS) might have existed in the right testis 6 years before being diagnosed with testicular cancer. Testicular germ cell tumors are the most common solid tumor among adolescent and young adult males.^3^ In the future, molecular biological experiments are expected to clarify the mechanism of testicular cancer carcinogenesis.

## Conflict of interest

The authors declare no conflict of interest.
